# A novel score to predict in-hospital mortality for patients with acute coronary syndrome and out-of-hospital cardiac arrest: the FACTOR study

**DOI:** 10.1007/s00392-023-02367-1

**Published:** 2024-02-08

**Authors:** Victor Schweiger, Pauline Hiller, Rahel Utters, Angela Fenice, Victoria Lucia Cammann, Davide Di Vece, Katja Rajman, Alessandro Candreva, Alexander Gotschy, Thomas Gilhofer, Michael Würdinger, Barbara E. Stähli, Burkhardt Seifert, Stefan M. Müller, Christian Templin, Julia Stehli

**Affiliations:** 1 Department of Cardiology, University Heart Centre, University Hospital Zurich, University of Zurich, Raemistrasse 100, 8091 Zurich, Switzerland; 2 Division of Biostatistics, Epidemiology, Biostatistics, and Prevention Institute, University of Zurich, Raemistrasse 100, 8091 Zurich, Switzerland; 3 Schutz & Rettung Zürich, Neumühlequai 41, 8021 Zurich, Switzerland

**Keywords:** Out-of-hospital cardiac arrest, Resuscitation, Acute coronary syndrome

## Abstract

**Introduction:**

Acute coronary syndromes (ACS) represent a substantial global healthcare challenge. In its most severe form, it can lead to out-of-hospital cardiac arrest (OHCA). Despite medical advancements, survival rates in OHCA patients remain low. Further, the prediction of outcomes in these patients poses a challenge to all health care providers involved. This study aims at developing a score with variables available on admission to assess in-hospital mortality of patients with OHCA undergoing coronary angiography.

**Method:**

All patients with OHCA due to ACS admitted to a tertiary care center were included. A multivariate logistic regression analysis was conducted to explore the association between clinical variables and in-hospital all-cause mortality. A scoring system incorporating variables available upon admission to assess individual patients' risk of in-hospital mortality was developed (FACTOR score). The score was then validated.

**Results:**

A total of 291 patients were included in the study, with a median age of 65 [56–73] years, including 47 women (16.2%). The in-hospital mortality rate was 41.2%. A prognostic model was developed in the derivation cohort (*n* = 138) and included the following variables: age, downtime, first detected rhythm, and administration of epinephrine. The area under the curve for the FACTOR score was 0.823 (95% CI 0.737–0.894) in the derivation cohort and 0.828 (0.760–0.891) in the validation cohort (*n* = 153).

**Conclusion:**

The FACTOR score demonstrated a reliable prognostic tool for health care providers in assessing in-hospital mortality of OHCA patients. Early acknowledgement of a poor prognosis may help in patient management and allocation of resources.

**Graphical abstract:**

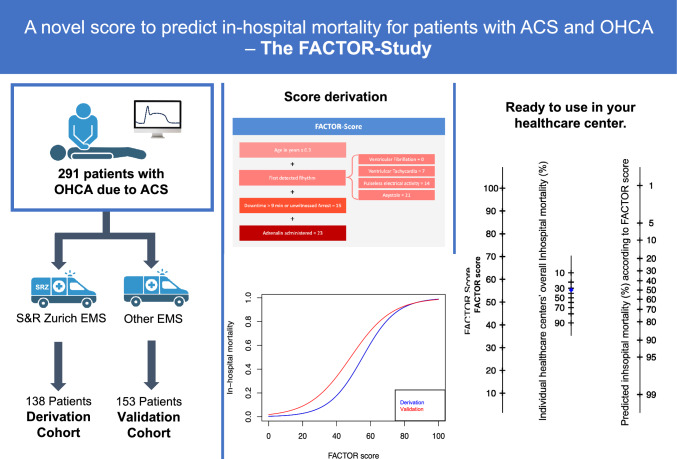

## Introduction

Coronary artery disease (CAD) stands as a prominent global healthcare challenge with an estimated death toll of 9.400.000 persons per year [1]. In its acute and most severe manifestation, CAD can culminate in acute coronary syndrome (ACS) and out-of-hospital cardiac arrest (OHCA). OHCA represents a considerable burden of medical emergencies throughout the world with an estimated incidence of 84 confirmed cases per 100.000 inhabitants per year in Europe alone and a potentially much higher number of unreported cases [2]. Regrettably, a substantial proportion of OHCA patients do not survive until hospital admission despite early cardiopulmonary resuscitation (CPR), with approximately 64% succumbing to the condition before arrival [2]. For those who survive until hospital admission, the mortality rate stays alarmingly high in the acute phase [3–5]. Despite medical advancements, the survival rates of OHCA patients did not significantly improve over the past 20 years, emphasizing the challenges with this patient cohort [3, 5].

Neurologic status plays a crucial role in the overall prognosis and potential for recovery in OHCA patients. Even with adequate CPR, brain perfusion remains subpar with only ~ 25% of the normal cerebral blood flow during cardiac arrest, posing the brain at high risk for permanent damage [6]. As a result, even professional CPR if performed over a long period and/or return of spontaneous circulation (ROSC) does not necessarily translate into favorable outcomes [6]. Thus, accurately predicting the prognosis of patients experiencing OHCA remains a multifaceted task, impacted not only by the cardiac condition but predominantly by the permanent neurological damage. However, accurate prognostication of neurological outcomes, and subsequently overall mortality, is crucial for effective resource allocation and the customization of treatment plans throughout the patient's hospitalization.

The present study, therefore, aims at developing and validating a predictive scoring system that combines variables available at the very beginning of admission to the emergency room for the specific population of patients with OHCA due to ACS undergoing coronary angiography. This will allow clinicians to reliably distinguish patients with a high likelihood of in-hospital mortality versus those who are likely to show recovery. By implementing such a scoring system, healthcare providers can strategically allocate resources and deliver targeted interventions to OHCA patients, ultimately improving patient outcomes and optimizing resource utilization.

## Methods

### Study design and data collection

**Fig. 1:**
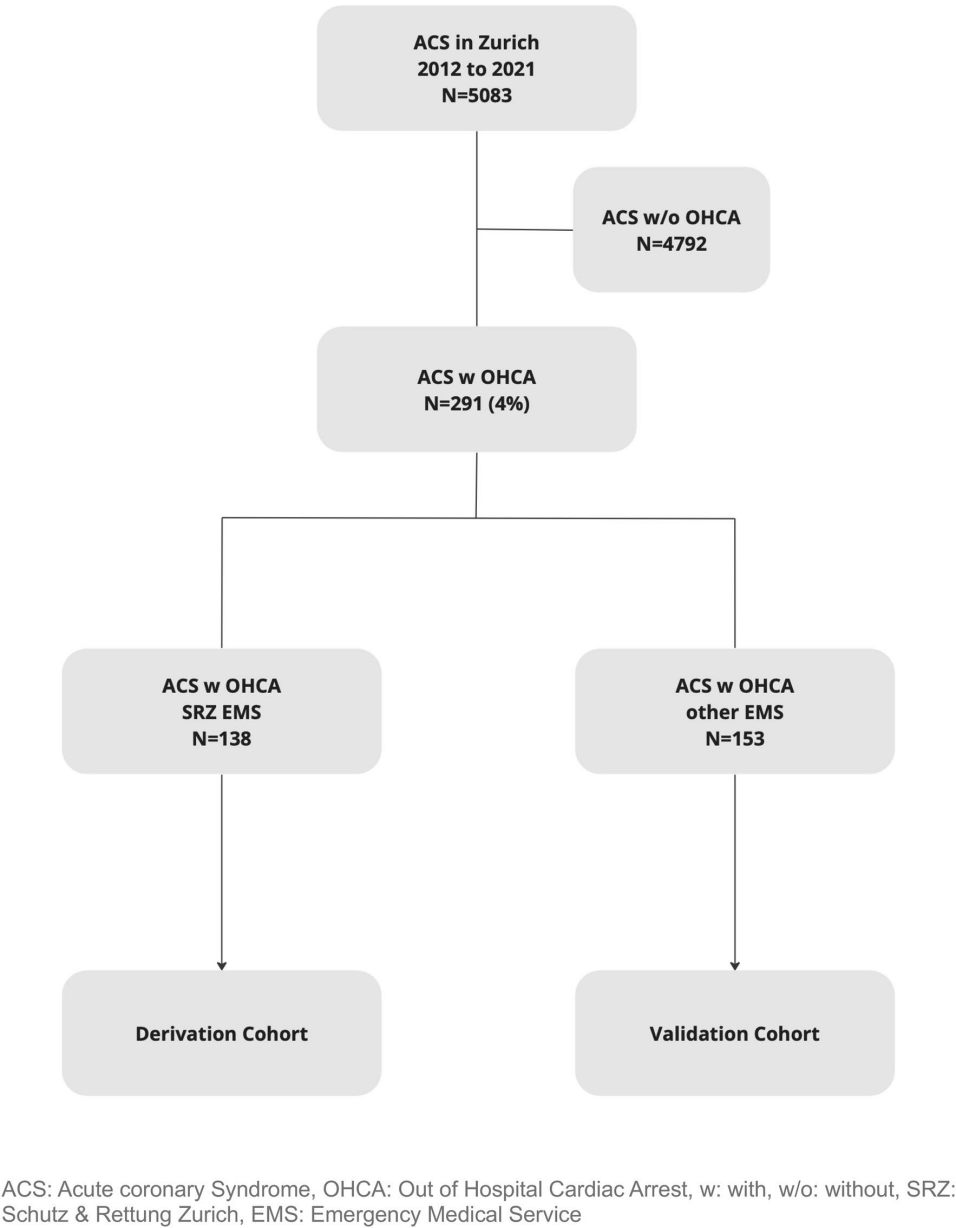
Flow chart of the study population

Clinical data of all OHCA patients who survived transport to the University Hospital of Zurich and who underwent coronary angiography for ACS between 01.01.2012 and 31.12.2021 were prospectively entered into a dedicated registry. ACS was defined according to the findings in the coronary angiogram. Resuscitation data (data about the time from cardiac arrest to arrival at the emergency department) were acquired directly from the local emergency medical service (EMS) facilities and transmitted into the database. The majority of EMS patients were transferred to the hospital by “Schutz & Rettung Zuerich” (SRZ), which is one of the largest EMS in Switzerland [7]. SRZ provides a 24-h emergency service with specially trained paramedics. All other patients were transferred by other EMS such as Schweizerische Rettungsflugwacht/Garde aérienne (Rega) or Alpine Air Ambulance (AAA), both Switzerland-based EMS services that use helicopters for transportation. For the score, patients transferred by SRZ were used as derivation cohort, whereas patients from all the other EMS services were used as validation cohort (Fig. 1).

## Definition of time intervals

### Downtime

Downtime was defined as the timespan from OHCA to the beginning of CPR through the first person at the scene. For calculation of the score, a downtime of ≥ 10 min was considered equal to an unwitnessed OHCA. A value of ≥ 10 min was chosen according to previous studies reporting on downtime and its influence on outcomes in OHCA [8].

### Time to ROSC

Time to ROSC was defined as the timespan from the beginning of CPR to ROSC.

### Total time to ROSC

Total time to ROSC was defined as the timespan from the OHCA to ROSC (sum of downtime and ‘time to ROSC’).

### Endpoints

The primary outcome was in-hospital all-cause mortality. The secondary outcome was a composite of in-hospital all-cause mortality and moderate or severe hypoxic encephalopathy. The latter was defined according to clinical judgement, which included symptoms, results from electro-encephalogram, neuron-specific enolase values, and magnetic resonance tomography if available**.**

### Statistical analysis

The distribution of variables was assessed graphically. Accordingly, continuous variables were described as median with interquartile range [IQR]. Groups were compared using the Mann–Whitney *U* test. Categorical variables were reported as frequencies and percentages and analyzed using Fisher’s exact test. Clinically selected variables were tested for their association with the primary outcome in the derivation cohort. Variables with a p-value < 0.20 were included in binomial multivariable logistic regression analyses. Model fit was assessed using the Hosmer–Lemeshow goodness-of-fit test. The best subset of variables using the Akaike information criterion (AIC, R package: bestglm) was used to derive the FACTOR score. Odds ratios (OR) with 95% confidence intervals and p-values were obtained individually for each of the clinical variables and for the score. The 95% confidence intervals of AUCs were computed using 2000 stratified bootstrap replicates. The score was then further validated in the validation cohort. To examine the performance of the FACTOR score in women, sex-specific ROC curves were compared using the pROC package in R. A two-sided p-value ≤ 0.05 was considered statistically significant. R version 4.2.1 (R Foundation, Vienna, Austria) was used for the statistical analyses and the compilation of graphs.

### Missing values

Missingness of data was assessed to determine their nature (not missing at random, missing at random, missing completely at random). A substantial proportion of the missing values were found to be not missing at random, therefore a clinical discussion was conducted, leading to the modifications of the variable: “Downtime (≥ 10 min) or unwitnessed arrest”. For the variables subjected to multivariable analysis, only a minimal amount of missing data was observed, thus they were imputed using their respective median values. The extent of missing variables can be found in Supplementary Table 4. Notably, no missingness was identified in the variables included in the scoring system.

### Ethics statement

The study was conducted according to the ethical principles of the Declaration of Helsinki. The local ethics committee reviewed the study protocol (BASEC-ID: 2018–02121). To be included in this study, patients or their relatives were asked for their consent to participate prior to inclusion. For deceased patients or those included in the study before 2016, the relevant ethics committee waived the requirement to obtain informed consent.

## Results

### Baseline characteristics

Between January 2012 and December 2021, a total of 5083 patients with ACS presented to the University Hospital Zurich. Out of these, a total of 291 consecutive individuals presented with OHCA and underwent coronary angiography. PCI was performed in 274 cases, referral to CABG in 9 of cases and no further intervention was done in 8 patients. Of the total cohort, 138 (47.4%) patients were included in the derivation cohort and 153 (52.6%) patients in the external validation cohort (Fig. 1). Table 1 shows the baseline characteristics: The median age was 65 years [56 to 73]. The proportion of females overall was 16.2%. Hypertension was present in 48.1% of the patients, while 17.5% had diabetes. 4.2% of patients had a history of myocardial infarction. Suppl. Table 1 displays the patients characteristics stratified by derivation and validation cohort.

**Table 1:** Baseline characteristics overall and stratified by survival

Patient characteristics	Overall	Survivors	Non-Survivors	
Baseline variables	*N* = 291	*N* = 171	*N* = 120	*p*-value
Age (years), *median (SD)*	65.00 [56.50, 73.00]	68.00 [59.00, 76.25]	61.00 [55.00, 69.50]	< 0.001
Female,* N (%)*	47 (16.2)	22 (12.9)	25 (20.8)	0.098
ACS,* N (%)*				0.845
STEMI	248 (85.2)	145 (84.8)	103 (85.8)	
NSTEMI	43 (14.8)	26 (15.2)	17 (14.2)	
BMI, *median (SD)*	26.20 [24.40, 29.30]	26.10 [23.85, 28.50]	26.30 [24.80, 29.40]	0.104
Hypertension*, N (%)*	140 (48.1)	73 (42.7)	67 (55.8)	0.034
Diabetes mellitus,* N (%)*	51 (17.5)	24 (14.0)	27 (22.5)	0.085
Smoking status, *N (%)*	142 (49.1)	84 (49.2)	58 (48.3)	0.671
Hypercholesterolemia, *N (%)*	77 (26.5)	2 (1.2)	3 (2.5)	0.093
Family history of ACS, *N (%)*	52 (17.9)	34 (19.9)	18 (15.0)	0.365
History of stroke, *N (%)*	3 (1.0)	2 (1.2)	1 (0.8)	1.000
History of myocardial infarction,* N (%)*	12 (4.1)	9 (5.3)	3 (2.5)	0.388

### Course of resuscitation and clinical variables on admission

Table 2 summarizes the most important datapoints from resuscitation until clinical variables on admission: The median downtime was 1 min [0 to 5]. Median time to ROSC was 15 min [8 to 25]. In 56.7% CPR was initiated by non-professionally bystanders, in 10% by policemen or firefighters and in 10% by medical personal who were not EMS paramedics. In the remaining 23.3%, CPR was initiated by professionally trained EMS paramedics. Ventricular fibrillation was the most commonly observed initial cardiac rhythm, occurring in 75.6% of patients. There was a significant disparity in the initial detected rhythm between survivors and non-survivors, with non-shockable rhythms being more commonly observed among non-survivors (*p* < 0.001). Median pH was 7.25 [7.16 to 7.32] and median lactate was 4.00 mmol/l [2.20 to 7.00]. Suppl. Table 2 displays procedural and clinical variables of patients stratified by derivation or validation cohort.

**Table 2:** Procedural and clinical variables of patients overall and stratified by survival

Clinical characteristics	Overall	Survivors	Non-Survivors	*p*-value
Procedural and clinical variables	*N* = 291	*N* = 171	*N* = 120	
Total downtime (min), *median [IQR]*	1:00 [0:00, 5:00]	0:00 [0:00, 5:00]	5:00 [0:00, 10:00]	< 0.001
Time to ROSC (min), *median [IQR]*	15:00 [8:00, 25:00]	12:50 [5:00, 22:00]	20:00 [15:00, 30:00]	< 0.001
Total time to ROSC (min),* median [IQR]*	20:00 [10:00, 30:00]	16:00 [8:00, 25:00]	28:00 [20:00, 38:00]	< 0.001
First rhythm, *N (%)*				< 0.001
Ventricular fibrillation	220 (75.6)	146 (85.4)	74 (61.7)	
Ventricular tachycardia	13 (4.5)	10 (5.8)	3 (2.5)	
Pulseless electrical activity	32 (11.0)	10 (5.8)	22 (18.3)	
Asystole	26 (8.9)	5 (2.9)	21 (17.5)	
CPR initiated by, N (%)				< 0.001
SRZ	68 (23.3)	43 (25.1)	25 (20.8)	
Layperson	165 (56.7)	85 (49.7)	80 (66.7)	
Police/firefighters	29 (10.0)	15 (8.8)	14 (11.7)	
Medical personnel	29 (10.0)	28 (16.4)	1 (0.8)	
pH,* median [IQR]*	7.25 [7.16, 7.32]	7.29 [7.20, 7.34]	7.21 [7.09, 7.30]	< 0.001
NSE (ng/ml), *median [IQR]*	18.40 [14.02, 22.05]	15.75 [12.88, 19.15]	66.55 [43.27, 89.82]	0.096
NSE max (ng/ml), *median [IQR]*	30.20 [17.85, 76.48]	19.15 [15.75, 26.60]	73.50 [37.70, 146.12]	< 0.001
Lactate (mmol/l),* median [IQR]*	4.00 [2.20, 7.00]	2.95 [1.83, 5.88]	5.85 [3.18, 7.80]	< 0.001
Lactate max (mmol/l), *median [IQR]*	4.60 [2.60, 7.70]	3.60 [2.10, 5.97]	6.20 [4.53, 9.20]	< 0.001
Creatinine (µmol/l), *median [IQR]*	107.00 [95.00, 127.00]	101.00 [87.00, 117.00]	118.50 [103.75, 135.00]	< 0.001
GFR (ml/min),* median [IQR]*	59.00 [47.00, 73.00]	64.00 [53.00, 77.50]	50.00 [40.75, 60.00]	< 0.001
Hemoglobin (g/l),* median [IQR]*	140.00 [127.00, 150.25]	140.00 [128.00, 150.50]	139.00 [125.00, 150.00]	0.314
Troponin T (ng/l)*, median [IQR]*	273.50 [82.00, 968.00]	234.50 [79.25, 841.25]	366.00 [100.50, 1224.50]	0.071
proBNP (ng/l),* median [IQR]*	796.00 [219.00, 2215.00]	556.50 [201.25, 1757.75]	1168.00 [323.00, 3644.00]	0.002
White blood cells count (G/l)*, median [IQR]*	14.29 [10.41, 17.88]	13.17 [9.67, 16.50]	15.93 [12.12, 19.48]	< 0.001
GCS at hospital admission,* median [IQR]*	3.00 [3.00, 3.00]	3.00 [3.00, 4.00]	3.00 [3.00, 3.00]	< 0.001
Shocks administered (overall)*, median [IQR]*	2.00 [1.00, 4.00]	2.00 [1.00, 4.00]	2.00 [1.00, 5.00]	0.678
Shocks administered by laypersons, *median [IQR]*	0.00 [0.00, 0.00]	0.00 [0.00, 1.00]	0.00 [0.00, 0.00]	0.001
Norepinephrine (µg)*, median [IQR]*	0.00 [0.00, 0.00]	0.00 [0.00, 0.00]	0.00 [0.00, 10.00]	< 0.001
Administration of epinephrin, *N (%)*	176 (60.5)	74 (43.3)	102 (85.0)	< 0.001
Glucose (mmol/l*), median [IQR]*	9.51 (3.59)	9.04 (2.90)	10.15 (4.30)	0.048
SO2 (%)*, median [IQR]*	91.00 [80.00, 97.00]	93.00 [85.00, 97.50]	86.00 [78.00, 94.00]	< 0.001
CO2 (mmHg)*, median [IQR]*	29.00 [18.80, 35.00]	29.00 [22.00, 35.00]	24.45 [10.68, 34.50]	0.147

### Outcomes

Out of the total 291 patients, 120 died within the hospital stay, representing an in-hospital all-cause mortality of 41.2%. The mortality was 33.3% in the derivation cohort and 48.4% in the validation cohort. The median length of hospital stay was 9 days [5 to 17], and the median survival time in the hospital was 5 days [2 to 8]. Overall survival with good neurological outcome was present in 55.6% of patients (63.0% in the derivation and 49.0% in the validation cohort. Any degree of hypoxic encephalopathy was significantly associated with the occurrence of in-hospital death (OR: 6.8, und *p* < 0.001). The association was even more pronounced in moderate or severe hypoxic encephalopathy (OR: 24.1, *p* < 0.001).

### FACTOR score

**Fig. 2:**
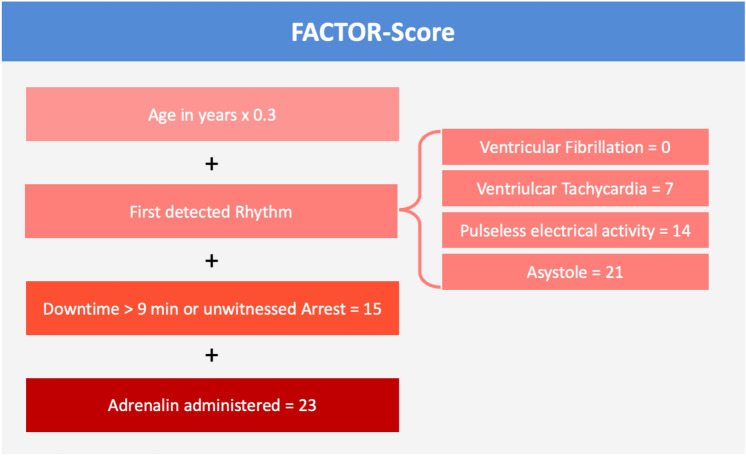
Variables used for the calculation of the FACTOR score. The clinical variables are weighted according to their impact on in-hospital mortality with corresponding points for each variable

**Fig. 3:**
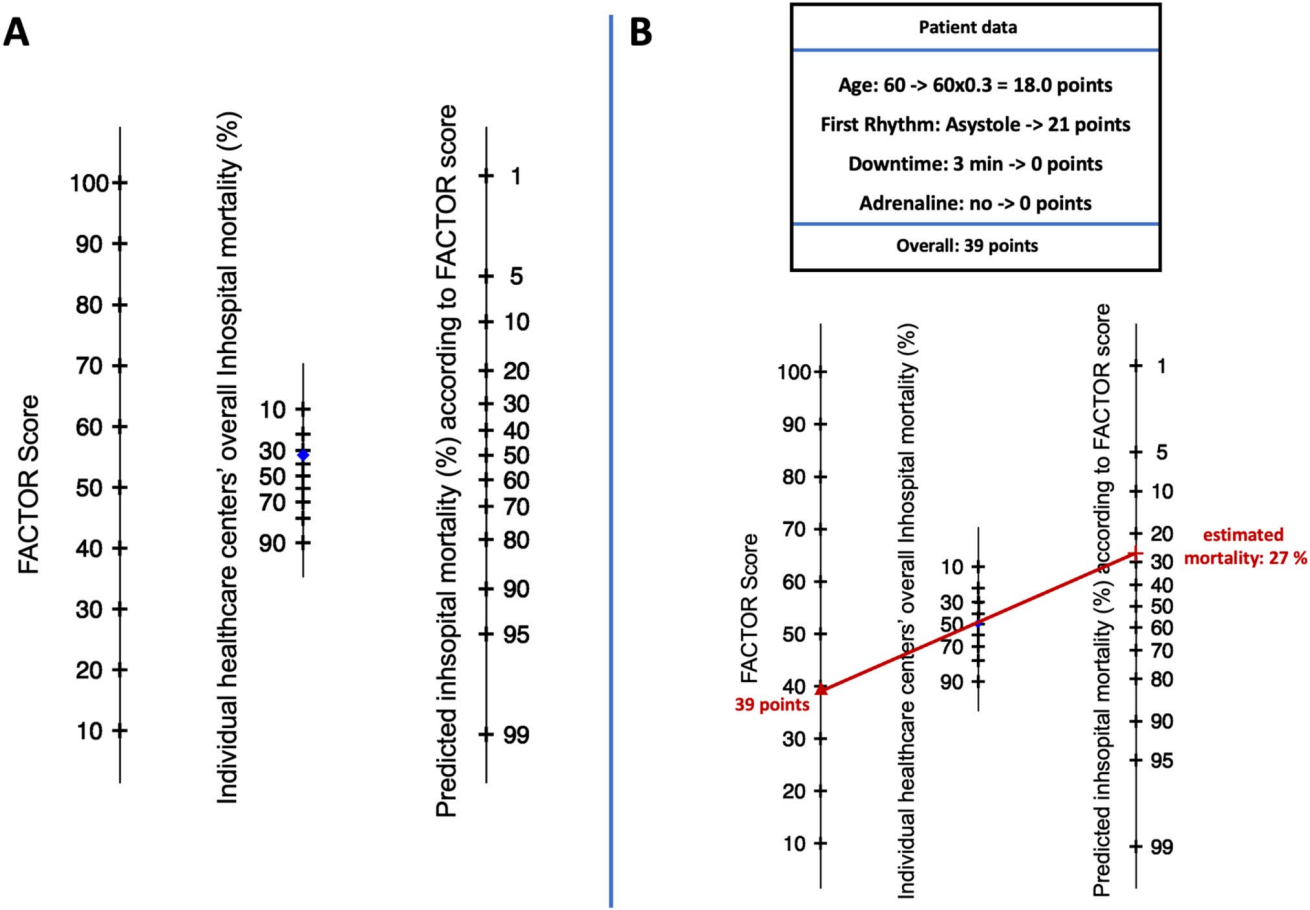
Nomogram to assess the individual patient’s risk based on the FACTOR score and pre-test probability of mortality (**A**). An example of estimating the mortality rate in a patient within a healthcare setting with an overall mortality rate of 50% (**B**).

To explore the association between clinical variables and the endpoint of in-hospital all-cause mortality, a scoring system was developed (FACTOR score). It incorporates variables available upon the patient’s admission to assess each patients' risk of in-hospital mortality. The score was developed from the derivation cohort, which comprises all patients transported to the hospital by SRZ EMS. The univariable analyses of the association of clinical variables with the endpoint is displayed in Suppl. Table 3. The final binomial multivariable logistic regression model (Table 3) was used to develop the score and included the following variables as main predictors for in-hospital mortality: age, first detected rhythm, downtime (≥ 10 min or unwitnessed arrest), and the administration of epinephrine. The score can be derived by multiplying the variables with the corresponding coefficients. For convenience, the coefficients were multiplied by 10 and rounded. This simplified way to calculate the score is illustrated in Fig. 2. The probability of in-hospital mortality can then be calculated by: 1 / (1 + exp (-intercept—0.1*score) or more easily by reading the nomogram (Fig. 3 A and B).

**Table 3:** Multivariable logistic regression analysis including the intercept from the derivation cohort

Multivariable analysis			
Variables	Coefficient	Std. Error	*p*-value
Intercept	− 6.37328249	1.6209308	< 0.001
Age	0.03295285	0.0192188	< 0.001
Downtime	1.47746207	0.494928	< 0.001
First detected rhythm	0.67971713	0.3847862	< 0.001
Administration of epinephrin	2.30238507	0.5594822	< 0.001

**Fig. 4:**
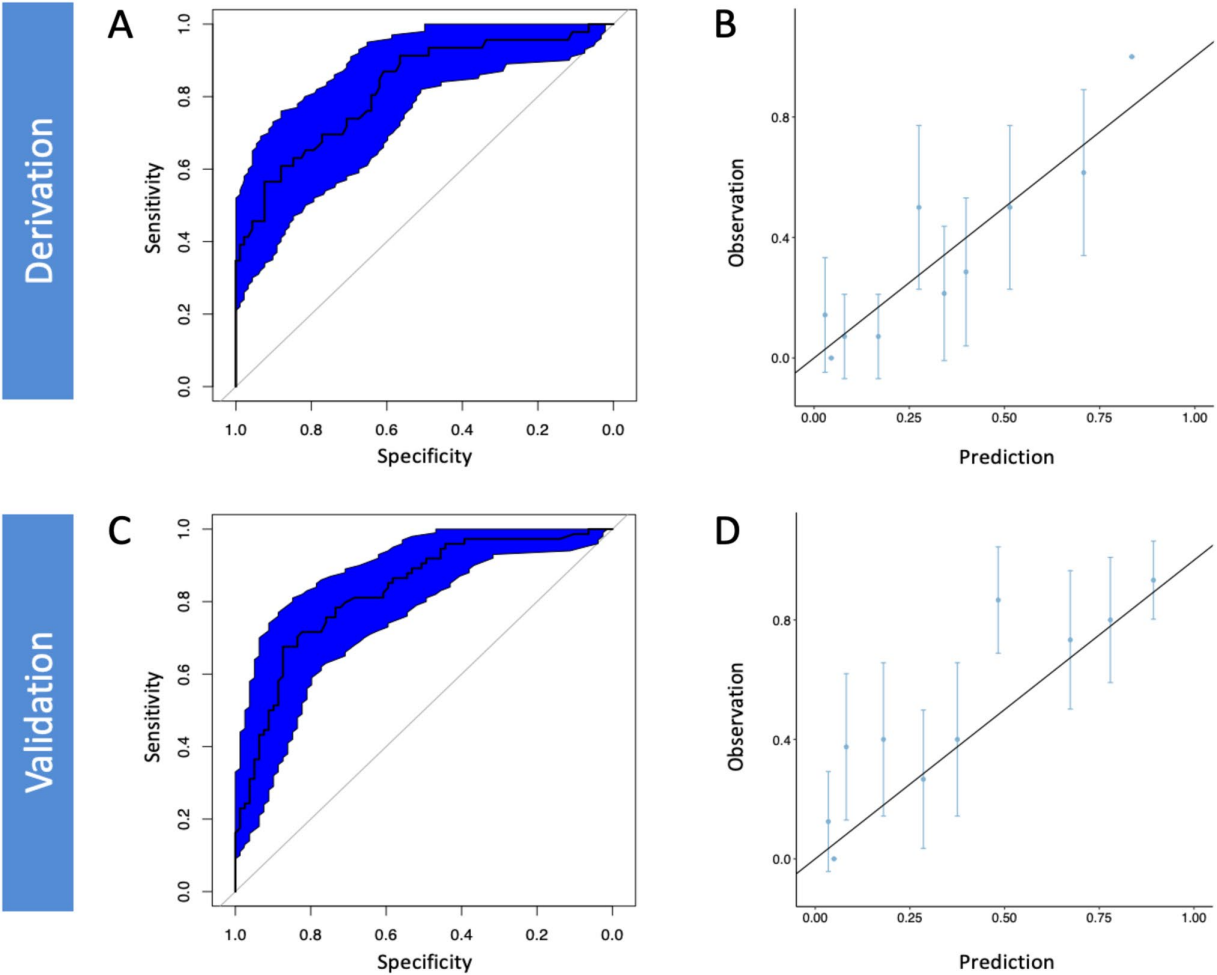
AUCs with 95% confidence intervals (**A**, **C**), calibration plot of the FACTOR score in the derivation and validation cohort (**B**, **D**)

**Fig. 5:**
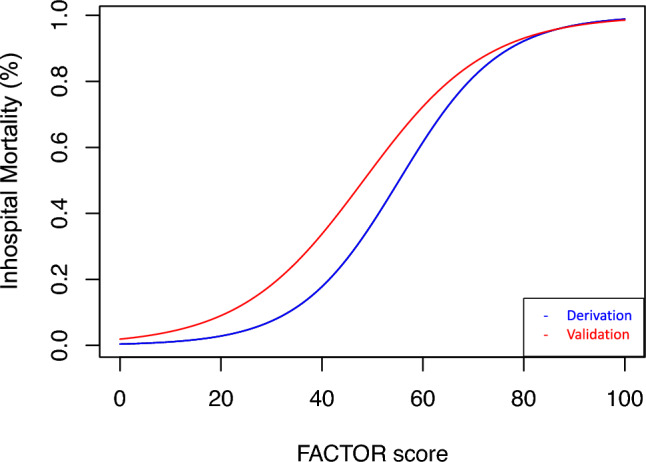
Risk estimation based on the FACTOR score

The score demonstrated an AUC of 0.82 (95% CI: 0.74–0.89) in the derivation cohort (Fig. 4A). The Hosmer–Lemeshow test had a p-value of 0.154, showing a good fit of the model. Calibration of the score was also good, as depicted by the respective calibration plot (Fig. 4B). The likelihood of in-hospital mortality based on the corresponding risk score estimate for the derivation and the validation cohort is displayed in Fig. 5**.**

In the external validation cohort, the score demonstrated an AUC of 0.83 for the primary endpoint of in-hospital all-cause mortality (95% CI: 0.76–0.89, Fig. 4C). Calibration of the score was satisfactory in the validation cohort, as depicted by the respective calibration plot (Fig. 4D). Regarding the secondary endpoint the score achieved an AUC of 0.82 in the validation cohort (95% CI: 0.75–0.88).

In terms of the sex-specific predictive performance of the score for the primary endpoint in the validation cohort, the AUC with a 95% CI was 0.81 (0.66–0.96) for women and 0.83 (0.76–0.91) for men (*p* = 0.78), indicating no statistically significant difference between the two groups.

The predicted probability is inherently linked to the overall in-hospital mortality rate of the treating healthcare center. Consequently, when utilizing the score in another setting with different mortality rates, it is imperative to appropriately adjust the conditional odds **(**Fig. 3A). This adjustment ensures the accuracy and relevance of the score's applicability in various diverse healthcare environments. Figure 3B depicts an example of the scores mortality estimation in a patient and a healthcare setting with an overall mortality rate of 50%.

## Discussion

The present study aimed at developing and externally validating a scoring system for the prognostication of in-hospital all-cause mortality of patients with OHCA undergoing coronary angiography. By utilizing multivariable logistic regression analysis, key variables that are associated with increased in-hospital mortality were identified and incorporated into an easily computable score (FACTOR score). External validation of the score confirmed its excellent prognostic accuracy. As such, a reliable prognostic tool was developed.

The FACTOR score utilizes only four clinical variables, explicitly: age, downtime (≥ 10 min or unwitnessed arrest), first detected rhythm and administration of epinephrine, all of which are readily available at the time of hospital admission. Despite using only four easily available variables, the FACTOR score achieved a reliable prediction of in-hospital mortality with an AUC of 0.83 in an external validation cohort. Furthermore, the FACTOR score demonstrated high diagnostic accuracy in predicting survival with good neurologic outcome in the external validation cohort.

Predicting outcomes is relatively straightforward when dealing with extreme cases in the clinical spectrum, such as those with very favorable or very unfavorable conditions. However, a majority of patients are found to be in a 'gray area,'. These unresponsive patients may undergo significant recovery despite initially inconclusive examination results even after a prolonged duration or they may never recover from the neurological damage [9]. So far, clinical tests are lacking accuracy. A score which could provide further help in prognostication would, therefore, be highly useful. Currently, guidelines advise waiting a minimum of 72 h post-cardiac arrest before conducting the initial neurological assessment without sedation, yet this duration might be too short [10]. It would, therefore, necessitate an extensively longer stay at the intensive care, leading to significant costs [9]. The FACTOR score could prove useful in allowing healthcare professionals in resource allocation in these specific settings.

Previous studies have proposed various scores (e.g., OHCA score and ACLS score) for prognostication in OHCA patients [3, 11–17]; however, they were mostly not externally validated [11–15], meanwhile outdated [13, 15, 16], incorporated only a small number of patients with OHCA due to cardiac causes or are very cumbersome to calculate [11, 12, 16]. Further, one score required an exact downtime, which is often not available, thereby excluding patients with unwitnessed cardiac arrest [16].

Another score to predict short-term mortality in OHCA patients is the NULL-PLEASE score. Even though it was derived in an only small patient cohort, it showed very good prognostic accuracy in an external validation study comprising a cohort of 700 patients with OHCA. However, its prognostic accuracy did not translate effectively to our cohort, only achieving an AUC of 0.59. Similarly, the OHCA score derived by Adrie et al. in a similar patient setting only achieved an AUC of 0.56 in our cohort [16]. This disparity in prognostic performance could be attributed to several factors. For instance, differences in the prevalence of CAD, as well as disparities in patient demographics and characteristics such as a lower prevalence of metabolic syndromes in Switzerland could have contributed. Further, differences in healthcare systems and EMS strategies could play a role. Another significant aspect that might influence the accuracy of the previously discussed scores in our cohort could be differences in geographical conditions: the present study was conducted in Switzerland, which has a dense population, thereby short transportation times and a possibly higher chance of bystander CPR. Moreover, it is among the regions with the highest density of hospitals and has a highly elaborated healthcare system. All together, these factors may also contribute to the relatively high number of survivors compared to other studies. However, the majority of the discussed studies did not display important results such as the baseline characteristics of their patient cohort and hence, our interpretations remain hypothesis generating only. Lastly, some scores such as the NULL-PLEASE score used cut off values for variables only. To address the lack of accuracy of previous published tests and enhance the applicability of our study, we developed a comprehensive nomogram within our patient cohort. This nomogram incorporates our scoring system along with the health care centers’ overall mortality rates for patients experiencing OHCA.

A strong association between neurological outcomes and mortality was observed. The most likely explanation is that there were more treatment discontinuations due to a limited neurological prognosis. Decisions on discontinuation of treatment, however, are also further influenced by other limiting comorbidities. However, given that the primary focus of this study did not involve predicting neurological outcomes, it is plausible that the development of a score specifically designed to predict favorable neurological recovery could potentially yield superior predictive performance. The main objective of the secondary endpoint was to offer insights into survival with a good quality of life.

Interestingly, the mortality risk in patients with CPR initiation by laypersons tended to be comparable to that of initiation by policemen or firefighters (Suppl. Table 3). However, the initiation of CPR by medical personnel that was on the scene by chance significantly reduced the risk of in-hospital death. The significant reduction in mortality after CPR by medical personnel compared to laypersons or semi-professionals (firefighters or policemen) suggests that if initial CPR is not performed through medical personnel, it is likely insufficient. Consequentially, mortality can be substantially reduced if CPR is sufficiently performed from the very beginning. This reflects the urgent need for enhanced CPR training in the general population as well as firefighters and police officers, who frequently initiate CPR (10% in this study). Ultimately, the implementation of enhanced CPR training and additional educational resources could yield favorable outcomes on a broader scale [18].

Sex differences in the prognostic accuracy of scoring systems can often be observed [19]. This might be attributable to the lower prevalence of female patients in studies involving patients with ACS and that while initially presenting similarly in the ER, women and men do have different outcomes [19, 20]. In the present study, when comparing the prognostic performance of the FACTOR score on the all-cause mortality between males and females, it was found that the FACTOR score exhibited similar diagnostic accuracy in both sexes. However, in the present studies’ cohort, female patients only comprised 16% of the total population.

Limitations.

Some limitations merit consideration. The study is limited by its retrospective, single-center design with a moderate sample size, which, however, represents a larger derivation cohort than the so far best validated Null-PLEASE score [21]. Further, since only patients who also underwent coronary angiography were included, a selection bias exists. We, therefore, cannot comment on patients who have had therapy withdrawn, while in the emergency department due to a dismal neurological prognosis.

Switzerland has a rather dense population and a well-developed health care system. Therefore, the applicability and usefulness of our score may be constrained when employed in a distinct patient population and healthcare system, as well as different geographical contexts such as rural areas.

## Conclusion

The proposed score, which was developed for OHCA patients with ACS who underwent coronary angiography, showed profound prognostic accuracy for in-hospital mortality. The variables included in the score are readily available at the arrival of the patient at the emergency room, thus providing clinicians with a valuable tool to estimate patient outcomes and allocate appropriate resources.

## Data Availability

The dataset associated with this study is available upon request under reasonable conditions.
